# The Rubber Case at Canandaigua N. Y.

**Published:** 1868-07

**Authors:** 


*The Rubber Case at Canandaigua N. Y.*—The following letter regarding this suit has been received from D. H. Porter of the Porter manufacturing Company: At the Untted States Circuit Court in session at Canandaigua N. Y. a motion was made by Chas. F. Blake Esq. counsel for the Hard Rubber Company, for an injunction against Dr. S. S. Smith of Syracuse, Dr. Hamess of Skaneateles, Drs. Mattson and Tripp of Auburn to restrain them from the further use of hard rubber for dental purposes. This motion was *successfully* opposed by W. H. Davis Esq. of Utica, counsel for the defendants, and the motion *denied* by the court in all cases with costs.

